# Actin Remodeling in Skeletal Myogenesis from Nuclear Signaling to Myoblast Fusion

**DOI:** 10.3390/cimb48101064

**Published:** 2026-10-15

**Authors:** Thanh Huu Phan Ngo, Wan Lee

**Affiliations:** 1 Department of Biochemistry, Dongguk University College of Medicine, 123 Dongdae-ro, Gyeongju 38066, Republic of Korea; thngo139@gmail.com; 2 Medical Research Institute, Dongguk University College of Medicine, 123 Dongdae-ro, Gyeongju 38066, Republic of Korea

**Keywords:** skeletal myogenesis, actin remodeling, mechanotransduction, MRTF–SRF, YAP/TAZ, myoblast fusion, satellite cells

## Abstract

Actin remodeling accompanies myoblast proliferation, lineage commitment, elongation, fusion, and myofibril assembly, but these changes do not constitute a single linear pathway. This review separates three experimentally distinguishable scales of actin function in skeletal myogenesis. First, the balance between monomeric and filamentous actin controls myocardin-related transcription factor–serum response factor (MRTF–SRF) signaling and thereby couples cytoskeletal state to gene expression. Second, integrin–focal adhesion kinase (FAK)–Rho signaling and actomyosin tension influence Yes-associated protein (YAP) and transcriptional coactivator with PDZ-binding motif (TAZ) activity, with outcomes that depend on cell state, density, mechanical context, and the non-equivalent functions of YAP and TAZ. Third, spatially restricted actin-related protein 2/3 (Arp2/3)-dependent branched actin networks generate protrusive force at the fusogenic synapse, while a mechanically reinforced receiving-cell cortex provides counterforce and cooperates with membrane fusogens to form and enlarge fusion pores. We compare evidence from cultured myoblasts, developmental models, and adult muscle regeneration, with attention to what each assay measures. We propose that bulk actin abundance, filament turnover, mechanical tension, and local actin architecture should be treated as separate variables. This framework reconciles apparently conflicting results and identifies experiments that can distinguish correlation from causal actin-to-nucleus signaling during muscle formation and repair.

## 1. Introduction

Skeletal muscle formation requires proliferating progenitors to withdraw from the cell cycle, activate a lineage-restricted transcriptional program, change shape, align with neighboring cells, and fuse into multinucleated fibers. The regulatory logic is usually described through myogenic transcription factors and extracellular signaling pathways. That description is necessary but incomplete because each transition also changes cell architecture and force transmission. Actin is therefore positioned both downstream of lineage signals and upstream of transcriptional and membrane-remodeling events. Developmental studies define the transcriptional sequence that produces myogenic cells, whereas culture and regeneration models expose the mechanical decisions made within that sequence [1,2].

The actin cytoskeleton is not a homogeneous pool. Monomeric globular actin (G-actin), linear and branched filament networks, cortical assemblies, contractile stress fibers, and sarcomeric thin filaments differ in location, lifetime, binding partners, and mechanical output. Actin-binding proteins further specify nucleation, elongation, severing, capping, crosslinking, and motor-driven contractility [3,4]. Measurements that collapse these structures into one F-actin signal can therefore obscure the relevant mechanism. A higher phalloidin intensity, for example, does not by itself establish faster polymerization, greater tension, or productive assembly at a fusion site.

This distinction matters because actin can regulate signaling through several routes. Changes in the available G-actin pool control myocardin-related transcription factors (MRTFs), actin-binding transcriptional coactivators whose release from G-actin permits nuclear accumulation and coactivation of serum response factor (SRF). In myogenic cells, MRTF–SRF activity supports cytoskeletal and muscle-gene expression and contributes to differentiation and muscle development [5,6,7]. Mechanical loading and actomyosin organization influence the Hippo effectors Yes-associated protein (YAP) and transcriptional coactivator with PDZ-binding motif (TAZ). At cell–cell contacts, actin-related protein 2/3 (Arp2/3)-dependent networks produce local protrusions that help overcome the energetic barrier to membrane fusion. In addition, actin organization changes receptor trafficking, adhesion maturation, nuclear shape, and the accessibility of signaling complexes. These routes can operate in the same cell but are not interchangeable, a point that is often lost when the actin cytoskeleton is treated as a single signaling entity [8,9].

A second source of confusion is that common myogenic models answer different questions. C2C12 cells are useful for pathway perturbation during a synchronized differentiation response, but they do not fully reproduce satellite-cell quiescence, a three-dimensional (3D) extracellular matrix, donor variability, or the asymmetric fusion behavior seen in vivo. Primary murine and human myoblasts provide closer species and donor relevance, although isolation activates the cells and extended culture on rigid plastic changes their state. Primary embryonic chicken myoblasts have been informative for myofibrillogenesis and YAP-dependent fiber-size control, whereas engineered 3D human muscle improves alignment, matrix geometry, and contractile maturation but does not reproduce the complete neural, vascular, immune, or systemic environment [10,11,12,13]. Drosophila embryos reveal the architecture of the fusogenic synapse at high spatial resolution, yet the identity and redundancy of nucleation-promoting factors differ from those in mammalian muscle. Adult injury models add a physiologic niche and immune response, but make it harder to separate direct effects on myoblasts from changes in inflammation, vascularization, or matrix remodeling. Evidence is strongest when a mechanistic claim survives more than one of these contexts.

The central question of this review is how actin organization is converted into distinct cell-fate outputs during skeletal myogenesis. We make three contributions. First, we separate global monomer–polymer balance, cell-scale tension, and contact-restricted branched actin as different causal variables. Second, we compare the MRTF–SRF and YAP/TAZ pathways without assuming that nuclear localization or total F-actin is sufficient evidence of transcriptional output. Third, we connect these nuclear pathways to the physical mechanics of fusion and identify experiments that can distinguish direct cytoskeletal signaling from correlated changes in differentiation. We discuss sarcomeric maintenance only where it clarifies the transition from myotube formation to mature muscle [14].

## 2. Literature Search Strategy

PubMed was searched using combinations of the terms skeletal myogenesis, myoblast, satellite cell, actin remodeling, mechanotransduction, MRTF, SRF, striated-muscle activator of Rho signaling (STARS), YAP, TAZ, focal adhesion, linker of nucleoskeleton and cytoskeleton (LINC) complex, nesprin, cell division cycle 42 (Cdc42), myosin II, spectrin, piezo-type mechanosensitive ion channel component 1 (PIEZO1), Arp2/3, neuronal Wiskott–Aldrich syndrome protein (N-WASP), WASP-family verprolin-homologous protein (WAVE), cadherin, catenin, desmin, microtubule, three-dimensional muscle, muscle atrophy, myonucleus, myoblast fusion, Myomaker, and Myomerger. Reference lists of relevant primary articles and focused reviews were used to identify earlier mechanistic studies. Selection emphasized experiments that perturbed a defined cytoskeletal or signaling component and measured a myogenic outcome. Publications were retained when they added a distinct mechanism or an in vivo test instead of merely repeating an association. The reference set and publication-status checks were updated through 5 October 2026.

This is a mechanism-oriented narrative review, not a systematic review or meta-analysis. No pooled effect size was calculated because models, mechanical environments, differentiation protocols, and readouts were heterogeneous. Evidence from non-muscle cells is used only to define a molecular mechanism that has subsequently been tested, or remains explicitly to be tested, in myogenic cells. Throughout, the wording distinguishes direct measurements from inferred quantities and identifies conclusions that remain dependent on a single cell line or perturbation method.

OpenAI ChatGPT (GPT-5.6 Sol, OpenAI, San Francisco, CA, USA) and Anthropic Claude (Opus 5.5, Anthropic, San Francisco, CA, USA) were used during the preparation and revision of this review as assistive tools for language editing, structural organization, preliminary figure-layout planning, and the identification and screening of candidate references. All references cited in added or revised passages were checked against the original publications. The authors independently determined source inclusion, evidence weighting, scientific interpretation, and the conclusions. They reviewed and approved all AI-assisted text and take full responsibility for the manuscript. The figures were drawn and edited by the authors in BioRender. The contribution of this review lies in its falsifiable organization of the evidence. It partitions actin function into three independently measurable scales, specifies what common assays do and do not establish, and proposes experiments that could support or refute the framework. The authors take responsibility for the framework and the predictions presented in Section 8.2.

## 3. Stage-Resolved Actin Remodeling During Myogenesis

### 3.1. A Changing Actin Architecture Across Myogenesis

Proliferating myoblasts use cortical actin, lamellipodia, and contractile bundles to spread, migrate, and sample their substrate. Cell-cycle withdrawal then reduces some growth-associated structures while enabling elongation and parallel alignment. Fusion introduces a different requirement. Actin must concentrate and remodel at selected cell–cell contacts without destabilizing the entire cortex. After fusion, actin increasingly supports myofibrillogenesis and sarcomere maturation. These events overlap, so the trajectory should not be represented as a simple rise or fall in total F-actin. The informative variable is actin organization at a defined place and time. Figure 1 summarizes these overlapping, stage-specific demands.

Pharmacologic manipulation of actomyosin contractility in skeletal myoblasts illustrates the separation between cell-cycle exit and terminal differentiation. Altering Rho-dependent contractility changed differentiation even when growth arrest was preserved, indicating that the two outputs are not obligatorily coupled [15]. This result does not mean contractility has a single direction of effect. The consequence depends on perturbation strength, timing, adhesion, and which actin network is affected. It does establish that cytoskeletal state contains regulatory information beyond the binary question of whether a cell has stopped dividing.

Elongation and alignment require coordination among actin, microtubules, and intermediate filaments. Non-muscle myosin II isoforms support the morphology changes that precede fusion, whereas the microtubule end-binding protein 3 (EB3) helps organize cortical dynamics needed for elongation and fusion [16,17]. In differentiating myoblasts, the M-cadherin–α/β-catenin complex associates with microtubules, providing a potential route by which cell–cell adhesion stabilizes longitudinal alignment [18]. Direct entry of microtubules into actin-rich filopodia is well suited to deliver membrane and polarity factors, but it has not yet been shown to be a necessary step in mammalian myoblast fusion. Desmin provides a complementary intermediate-filament scaffold, and suppression of desmin in C2C12 cells disrupts myogenic regulator expression and blocks fusion [19]. These findings argue for live, dual-color analysis of microtubule growth, actin protrusions, and desmin remodeling rather than attribution of every shape phenotype to actin polymer mass alone.

Mature muscle introduces another scale of actin control. Sarcomeric filaments are stabilized by a specialized protein ensemble, yet remain subject to length control and turnover. Cyclase-associated protein 2 (CAP2) deficiency delays differentiation of the myofibrillar actin cytoskeleton, and cofilin-2 loss causes abnormal actin accumulation or altered filament length in mouse muscle [20,21,22]. These phenotypes are important for muscle architecture but should not be used as direct evidence for the signaling mechanism that initiates myoblast differentiation. Early cell-fate control and late sarcomeric maintenance share molecules while imposing different spatial and mechanical demands.

The ratio between cytoplasmic and sarcomeric actin pools is not controlled by a single known rheostat. During myofibrillogenesis, cytoplasmic β/γ-actins and sarcomeric α-actins can enter nascent structures, but α-actin becomes preferentially incorporated into mature striated myofibrils [23]. The cytoplasmic pool is not merely residual, because muscle-specific deletion of cytoplasmic γ-actin in mice permits normal muscle development yet causes a progressive myopathy, which indicates that a minor, costamere-localized cytoplasmic pool carries a function that sarcomeric actin does not replace [24]. The ratio is therefore shaped by developmental actin-isoform expression, preferential incorporation into nascent myofibrils, stabilization by sarcomeric binding proteins, and turnover mediated by CAP and cofilin family proteins [20,21,22]. Soluble and cortical pools remain necessary for signaling, trafficking, repair, and fusion, so reduced cytoplasmic staining should not be interpreted as complete conversion into sarcomeric actin. Isoform-specific pulse–chase imaging combined with biochemical G/F fractionation would provide a quantitative test of exchange between these pools.

### 3.2. Matrix Mechanics and the Adhesion–Cytoskeleton Interface

Myogenic cells respond to the elastic and adhesive properties of their environment. C2C12 myotubes differentiate most effectively on substrates approximating muscle tissue with poorer differentiation on very soft or rigid materials [25]. Matrix elasticity also influences lineage choice in mesenchymal stem cells, although that broader result should not be equated with a muscle-specific mechanism [26]. For freshly isolated muscle stem cells, compliant culture substrates better preserve self-renewal and regenerative capacity than conventional rigid plastic [10]. Together, these studies show that substrate mechanics can change myogenic behavior, but they do not imply that a single stiffness value is optimal across developmental stage, species, ligand coating, and culture geometry.

Recent work with human myoblasts reinforces the need to measure both mechanics and cell state. Substrate stiffness altered contractility-dependent proliferation and nuclear-envelope wrinkling [11]. Nuclear morphology in this setting is a useful mechanical readout, but it does not directly report chromatin accessibility or transcription. Likewise, traction force or phosphorylated myosin light chain can support an inference about contractility, whereas phalloidin intensity alone cannot. Studies that combine substrate mechanics, traction or tension sensors, nuclear deformation, and transcript-level measurements are therefore more diagnostic than any one endpoint.

Nuclear shape must also be interpreted in light of the physical linkage between the cytoskeleton and the nuclear envelope. In migrating myoblasts, linker of nucleoskeleton and cytoskeleton (LINC) complex-mediated, actin-dependent nuclear positioning helps orient the centrosome [27]. In mouse skeletal muscle, loss of nesprin-1α2 disrupts myonuclear positioning and compromises postnatal viability [28]. These studies establish a muscle-relevant route for transmitting cytoskeletal organization to the nucleus, although nuclear wrinkling alone does not specify MRTF or YAP/TAZ activity.

Focal adhesions translate matrix engagement into biochemical and mechanical signals. Focal adhesion kinase (FAK) activity changes dynamically during C2C12 differentiation, and disrupting FAK signaling impairs the differentiation program [29]. Integrin–FAK signaling also cooperates with cell adhesion molecule-related/downregulated by oncogenes (Cdo), Cdc42, and myogenic differentiation 1 (MyoD) in a promyogenic pathway [30]. These results support a role for adhesion signaling, but FAK is not an actin-specific sensor. It also organizes growth-factor, survival, and polarity pathways. To causally attribute effects to actin, test whether restoring the relevant actin architecture or mechanical output rescues the adhesion phenotype without broadly reactivating FAK.

Mechanically gated ion channels provide another route from force to the cytoskeleton. During C2C12 myotube formation, piezo-type mechanosensitive ion channel component 1 (PIEZO1)-dependent calcium influx promotes RhoA–Rho-associated coiled-coil-containing protein kinase (ROCK) signaling and actomyosin assembly at the lateral cortex. This response limits uncontrolled fusion and supports polarized myotube elongation [31]. This finding shows that mechanical input can reorganize a spatially defined actomyosin network without first acting through a nuclear effector.

Mechanical inputs also differ in spatial extent and duration. In myoblasts, local repeated force and global strain produced distinct kinetics of actin remodeling and MRTF-A redistribution [32]. This is a general experimental lesson. A short, localized load at one adhesion is not equivalent to chronic culture on a stiff matrix. The former can create a transient actin response, whereas the latter also changes spreading, cell density, metabolism, and secreted matrix. Mechanotransduction studies should therefore report ligand identity, effective stiffness, cell geometry, confluence, loading waveform, and the interval between stimulation and measurement. Table 1 summarizes representative perturbation studies and their interpretive limits.

## 4. The Actin–MRTF–SRF Axis

MRTF proteins provide the most direct molecular link between the actin monomer pool and transcription. MRTF-A, originally termed MAL or megakaryoblastic leukemia 1 (MKL1), contains arginine-proline-glutamate-leucine (RPEL) motifs that bind G-actin. When the accessible G-actin pool is high, this interaction favors cytoplasmic retention and limits productive association with serum response factor (SRF). Actin polymerization reduces G-actin occupancy, enabling MRTF nuclear accumulation and coactivation of SRF-dependent genes [7]. Structural analysis of a pentavalent G-actin–MRTF-A complex explains how multiple actin molecules mask transport-related elements and provides a physical basis for this sensor [43].

The switch is more dynamic than a one-way cytoplasmic release mechanism. Actin regulates both MRTF-A nuclear import and export, and nuclear G-actin can bind MRTF-A, promote its export, and restrain its activity [44,45]. Nuclear G-actin is therefore a regulated signaling pool rather than passive spillover from the cytoplasm. Polymerization inside the nucleus is instructive rather than incidental, because formin-driven assembly of a transient nuclear actin network is required for serum-induced MRTF-A nuclear accumulation and SRF activity, and optogenetic release of formin autoinhibition drives that network reversibly [46]. Nuclear-envelope proteins add another layer. Lamin A/C and emerin modulate MKL1–SRF signaling through effects on actin dynamics, while lamina-associated polypeptide 2α supports intranuclear MRTF-A activity [47,48]. Direct quantitative mapping of nuclear G-actin and MRTF occupancy during myogenic progression remains limited. A stronger mechanistic chain would combine a nuclear actin-state sensor, G-actin binding or an actin-insensitive MRTF mutant, nuclear residence, SRF occupancy or reporter activity, target-gene expression, and a myogenic outcome.

The relevance of this axis to myogenesis was established before mechanobiology became a common framing. MRTF-A/MKL1 and MRTF-B/MKL2 are required for efficient skeletal myogenic differentiation in cultured cells [5]. The striated-muscle activator of Rho signaling (STARS/ABRA) promotes RhoA-dependent actin polymerization, MRTF nuclear accumulation, and SRF activity [34]. Earlier C2C12 work also showed that RhoA-dependent SRF activity supports skeletal alpha-actin expression, myogenin induction, and fusion [49]. Conditional loss of SRF in mouse skeletal muscle compromises postnatal growth and maturation [50]. These studies place SRF downstream of cytoskeletal signals but also show why pathway assignment requires care. SRF receives input from ternary-complex factors as well as MRTFs, and its targets include both structural and regulatory genes.

Genetic tests strengthen the conclusion that MRTFs are not redundant accessories. Combined loss of MRTF-A and MRTF-B in skeletal muscle produces hypoplastic muscle with disorganized sarcomeres and poor myoblast survival, culminating in perinatal lethality [6]. SRF also supports satellite-cell fusion by maintaining actin architecture in regenerating mouse muscle [51]. MASTR, also called MAMSTR, is a distinct SAP (SAF-A/B, Acinus, and PIAS)-domain coactivator of myocyte enhancer factor 2 (MEF2) and is not an MRTF family member. It promotes MyoD-dependent satellite-cell differentiation, but direct regulation by the G-actin–RPEL mechanism has not been established [52,53].

Upstream Rho signaling can produce different outputs. The RhoA effector mDia1 regulates MyoD and cell-cycle progression through both SRF-dependent and SRF-independent mechanisms [33]. Rho–ROCK can also induce inhibitor of DNA binding 3 (ID3) through MRTF-A/Smad signaling [54]. The effect of MRTF–SRF therefore depends on developmental stage, transcriptional partners, and target genes and cannot be inferred from polymerization alone.

Evidence from individual actin regulators is useful when it is interpreted at this level. WAVE2 perturbation in C2C12 cells links branched-actin regulation to MRTF-A localization, SRF activity, and myogenic differentiation [55]. The study supports an actin-sensitive transcriptional route in cultured cells, but WAVE-family proteins also regulate adhesion and membrane protrusion. Stage-restricted perturbation and MRTF epistasis are needed to determine which function dominates in satellite cells and regenerating muscle.

Metabolic and redox signals may converge on the same actin sensor. A recent study proposed that a reactive oxygen species (ROS)–dynamin-related protein 1 (Drp1)–mitophagy feedback loop influences differentiation through actin remodeling and the MRTF-A–SRF axis [56]. Because ROS has broad effects, the proposed ordering requires actin rescue, MRTF epistasis, and direct measures of mitochondrial quality. MRTF–SRF therefore functions as a sensor embedded within metabolism, adhesion, and nuclear mechanics and should not be treated as an isolated linear pathway.

Finally, MRTF abundance itself is regulated. Multiple microRNAs change MRTF-A expression during myoblast differentiation [57], and aged mouse muscle shows reduced expression of SRF and MRTF-A [58]. These observations identify potential control points but do not establish that lower MRTF activity is the primary cause of regenerative decline. Age changes satellite-cell composition, innervation, inflammation, matrix stiffness, and mechanical use. Cell-type-resolved and inducible genetic experiments are needed to separate a myogenic MRTF defect from secondary adaptation to an aged tissue.

## 5. Actomyosin Tension and YAP/TAZ Signaling

YAP and TAZ provide a second route from cytoskeletal mechanics to the nucleus. In many adherent cells, spreading, matrix stiffness, and actomyosin tension favor nuclear YAP/TAZ, whereas a compliant matrix or cytoskeletal relaxation favors cytoplasmic retention [59]. Actin-processing factors can also control YAP/TAZ through a mechanical checkpoint that is not fully explained by the canonical Hippo kinase cascade [60]. These foundational observations define a mechanosensitive system, but their direction cannot be transferred to myogenesis without accounting for lineage stage. In a proliferating myoblast, YAP/TAZ may sustain expansion. After commitment, prolonged activity can delay the same differentiation program needed to build muscle. PIEZO1–RhoA–ROCK signaling also reorganizes cortical actomyosin during myotube formation, but YAP/TAZ was not established as its downstream effector in that setting [31].

Actin microfilaments can also function as regulated binding platforms for Hippo pathway components rather than acting only through bulk tension. In non-muscle cells, angiomotin (AMOT) family proteins bind F-actin and YAP competitively, and their release from F-actin favors YAP retention and large tumor suppressor kinase (LATS)-dependent Hippo signaling [61]. AMOT proteins can therefore couple filament architecture to a signaling scaffold. This mechanism has not been directly established in myoblasts, so its muscle relevance requires demonstration of AMOT expression and localization, actin-dependent assembly of the AMOT–LATS–YAP complex, and a stage-specific myogenic phenotype.

In C2C12 cells, YAP promotes proliferation and inhibits terminal differentiation when maintained in an active state [62]. Satellite-cell experiments similarly place YAP at a fate decision. YAP activity supports progenitor expansion, whereas timely downregulation is required for differentiation [63]. These experiments directly implicate YAP, although TEA domain (TEAD)-dependent transcription, cell-cycle duration, and density-dependent feedback can still contribute to the observed outcome.

TAZ cannot be treated as a synonym for YAP in this context. TAZ can bind MyoD and enhance MyoD-dependent transcription and promote differentiation [64]. A direct comparison in muscle stem cells showed that YAP and TAZ both support proliferation, whereas TAZ also enhances later differentiation and fusion through TEAD4 [65]. Pharmacologic activation of TAZ likewise enhanced differentiation and regeneration in experimental models [66]. YAP and TAZ should therefore be measured and perturbed separately across the proliferation-to-differentiation transition.

Developmental studies further show that YAP output depends on mechanical use. In fetal muscle, contraction is required to maintain a progenitor pool through YAP and NOTCH signaling [67]. In differentiated fibers, Wnt/β-catenin signaling through Axin2 intersects with YAP/TAZ–TEAD activity in particular fiber types [68]. Experimental YAP activation can also change myofiber size in a protein kinase C-dependent manner during chick myogenesis [12]. These data extend YAP/TAZ beyond an early differentiation switch, but they describe distinct compartments, including fetal progenitors, mature fibers, and cultured embryonic muscle. Pooling them into one direction of effect would erase the biological question each model answers.

The apparent sign of YAP/TAZ activity is therefore a function of time, cell state, and endpoint. Nuclear YAP during an expansion phase can be compatible with successful regeneration if activity later falls. The same sustained signal during differentiation can be inhibitory. A larger fusion index can reflect more cells entering differentiation, better survival, or a direct change in membrane fusion. Conversely, lower expression of a differentiation marker can follow persistent proliferation without showing that YAP directly represses the marker. Time-resolved perturbations and lineage tracking are more informative than endpoint comparisons. Focused reviews of Hippo signaling in skeletal muscle reach the same general conclusion that pathway output is compartment- and context-dependent [69,70].

Actin regulators can alter YAP without being dedicated Hippo components. ROCK-dependent phosphorylation of calponin 3 (CNN3) contributes to fusion, and an independent knockdown study connected CNN3 to proliferation, differentiation, and mechanistic target of rapamycin (mTOR) signaling [71,72]. A C2C12 study also linked CNN3-dependent actin remodeling to YAP1 localization and myogenic outcomes [73]. MICAL2 is required for myogenic commitment, whereas MICAL1 perturbation in C2C12 cells altered actin organization, YAP1 localization, and differentiation [38,74]. Collectively, these studies identify cytoskeletal inputs to YAP but do not establish one exclusive pathway or transcriptional target.

The most useful synthesis is not that MRTF and YAP/TAZ report the same property of actin. MRTF responds directly to an available actin-monomer pool through RPEL binding, whereas YAP/TAZ integrate tension, adhesion, cell shape, polarity, and Hippo-dependent regulation. Polymerization and tension often covary, but they can be uncoupled. In cancer-associated fibroblasts, mutual dependence between MRTF–SRF and YAP–TEAD is indirect and mediated through cytoskeletal dynamics without evidence of direct binding [75]. This mechanism demonstrates feasible crosstalk but should not be assumed to operate unchanged in myoblasts. Simultaneous pathway reporters could determine whether the two systems interact or independently converge on the proliferation-to-differentiation transition.

## 6. Local Branched Actin at the Fusogenic Synapse

Myoblast fusion imposes a physical problem that is distinct from nuclear mechanotransduction. Cells must migrate into proximity, recognize an appropriate partner, align, assemble an adhesive contact, deform two cortices, initiate lipid mixing, and expand a fusion pore. Actin participates in several of these steps, so a lower fusion index after cytoskeletal perturbation is not sufficient to identify the affected one. Non-muscle myosin II and EB3 contribute to pre-fusion morphology and alignment [16,17], while Rac1, Cdc42, and the Rac activator Dock1 are required for efficient fusion in mice [39,40]. These early defects must be excluded before an actin regulator is assigned a direct role at the membrane-merger step.

Adhesive contacts organize this mechanical sequence rather than merely holding the cells together. M- and N-cadherin appear partly redundant in mammalian myoblasts, because mice lacking M-cadherin develop and regenerate muscle essentially normally with apparent compensation by N-cadherin [76], whereas disrupting cadherin surface localization inhibits C2C12 fusion without abolishing differentiation [77]. M-cadherin associates with α- and β-catenin and with microtubules in differentiating muscle cells, linking the junction to both actin and microtubule organization [18]. Importantly, suppressing canonical β-catenin signaling alone did not reproduce the fusion block, which argues that cadherin trafficking and junctional organization, rather than Wnt/β-catenin transcription alone, are central in this setting. Cadherin–catenin adhesion should therefore be analyzed together with actin protrusions, cortical resistance, and fusogen localization.

Drosophila studies established the concept of an asymmetric fusogenic synapse. Suppressor of cyclic AMP receptor/WASP-family verprolin-homologous (SCAR/WAVE) and Wiskott–Aldrich syndrome protein (WASP) family nucleation-promoting factors act in a defined sequence, and a dense actin focus forms at the contact between fusion-competent cells [78]. This network supports an invasive podosome-like structure that protrudes into the apposing cell and promotes fusion-pore formation [79]. Actin-propelled protrusions also promote engagement of fusogenic proteins at the contact [80]. Diaphanous-dependent linear actin and Arp2/3-dependent branched actin cooperate in this process, while phosphatidylinositol 4,5-bisphosphate helps localize Arp2/3 regulators at the fusion site [81,82]. The resulting model is spatial. A small, mechanically active actin focus is more relevant than the cell-wide F/G-actin ratio.

Subsequent cell-biological work mapped the spatiotemporal coordination of actin regulators that generates invasive protrusions during cell–cell fusion [83]. Simultaneous recruitment does not imply simultaneous function. Nucleation, network growth, turnover, and coupling to membrane proteins can be sequential even within a contact that lasts only minutes.

Force at the fusogenic synapse also depends on the receiving-cell cortex. In Drosophila, invasive protrusions trigger mechanosensitive recruitment of non-muscle myosin II in the receiving cell, which raises cortical tension and promotes fusion-pore formation [41]. Spectrin also accumulates in response to deformation, constrains the invasive protrusions, and increases local tension [42]. These studies define the synapse as a coupled system of protrusive and resisting forces. Whether mammalian fusion partners maintain the same stable asymmetry remains unresolved.

Mammalian experiments initially supported parts of this model through cultured-cell and developmental perturbations. Nap1-mediated actin remodeling is required for mammalian myoblast fusion [37]. Conditional deletion of neuronal Wiskott–Aldrich syndrome protein (N-WASP) in mouse myogenic cells preserved differentiation, motility, morphology, and initial cell–cell attachment but selectively impaired membrane fusion. Mixing experiments further indicated that N-WASP was required in both fusion partners [35]. Cyclase-associated protein 1 (CAP1) downregulation and Drebrin activity also support myotube formation, although their precise stage of action is less well resolved [84,85].

A 2026 conditional-knockout study resolves part of that ambiguity in adult mouse regeneration. Deleting Arp2/3 complex components or disrupting N-WASP and WAVE activity in satellite-cell-derived myoblasts reduced fusion and invasive protrusion formation, while proliferation, differentiation, and migration were comparatively preserved [36]. N-WASP and WAVE functions were partly redundant. This combination of stage-specific genetic perturbation, injury context, and direct morphology provides strong evidence that branched actin has a local, fusion-specific role in mammalian muscle repair. It does not prove that every detail of the Drosophila synapse is conserved, but it closes an important gap between the invertebrate structural model and mammalian physiology.

Actin-generated protrusion must ultimately cooperate with muscle-specific fusogens. Myomaker is required for myoblast fusion and muscle formation, whereas Myomixer/Myomerger confers complementary fusogenic activity and is required for skeletal muscle development [86,87,88]. The proteins act at distinguishable membrane-remodeling steps, with protrusive and resisting forces stressing apposed membranes and the fusogens altering lipid energetics [89].

Biophysical experiments support elastic coupling between proximal membrane leaflets and the hemifusion diaphragm during Myomerger-dependent pore formation [90]. Separate atomistic and coarse-grained molecular-dynamics simulations predict that Myomerger can promote hemifusion and regulate pore expansion [91]. Actin should therefore not be described as the fusogen. A defensible model combines local protrusive actin, receiving-cell resistance, fusogen-dependent membrane remodeling, and continued cytoskeletal force during pore growth.

The same regulator can operate at more than one scale. WAVE-family proteins can influence global actin balance and MRTF–SRF signaling during differentiation, yet they also contribute to local Arp2/3 activation at fusion contacts. Diaphanous can affect cell-cycle and MyoD regulation through SRF-related pathways and also organize actin during fusion. This is not mechanistic redundancy. It is reuse of an actin-control module in different subcellular compartments. Localization-specific perturbations, such as acute recruitment or inhibition at cell–cell contacts, are needed to assign the relevant function. Whole-cell knockdown alone cannot distinguish a nuclear-signaling defect from a local mechanical defect.

Each successful fusion event adds one or more nuclei to a growing syncytium, so actin-dependent contact formation and membrane merger influence the rate of myonuclear accretion. However, no evidence currently supports actin as a direct nucleus-counting system. Final myonuclear number is an emergent outcome of progenitor availability, fusion frequency and directionality, fusogen competence, fiber size, and activity-dependent demand. Titration of myonuclear number in three independent mouse models, which lowered myonuclei by 25 to 75 percent, showed that myonuclei retain a reserve capacity to support larger cytoplasmic volumes and that this reserve varies inversely with nuclear number [92]. Nuclear number therefore behaves as a limiting variable for developmental growth rather than a quantity that actin sets directly. Actin does, however, position nuclei once they are inside the syncytium. Actin–LINC coupling contributes to nuclear positioning [27,28], centripetal forces generated by contracting and crosslinked myofibrils move nuclei to the fiber periphery through an Arp2/3 complex containing Arpc5L together with γ-actin that organizes desmin [93], and N-WASP acts downstream of amphiphysin-2 in peripheral nuclear positioning and triad organization, with its distribution disrupted in centronuclear myopathy [94]. Single-nucleus transcriptomics has identified specialized myonuclear programs at neuromuscular and myotendinous junctions and in regenerating or dystrophic fibers [95]. These data establish transcriptional division of labor, but not that actin itself assigns nuclear identity. Acute spatial perturbation of actin combined with nuclear tracking and spatial or single-nucleus transcriptomics could test whether altered positioning changes nuclear specialization.

Fusion assays should therefore report more than a terminal fusion index. Cell number, viability, differentiation-marker expression, migration, alignment, contact duration, protrusion frequency, lipid mixing, pore opening, and pore expansion are separable outcomes. A perturbation that preserves Myogenin expression and migration but eliminates actin-rich protrusions supports a direct fusion mechanism more strongly than one that broadly reduces differentiation. Extending the conditional approach with tension sensors and endogenous fusogen tags could quantify how actin-generated force is coupled to the molecular machinery of membrane merger. Figure 2 integrates these local fusion mechanics with the two actin-to-nucleus signaling scales.

## 7. Regeneration, Aging, and Pathological Mechanical Contexts

Adult regeneration reuses much of the myogenic program within a mechanically changing tissue. Quiescent satellite cells activate beneath the basal lamina, proliferate, migrate through an injured environment, differentiate, and fuse with one another or with existing fibers. Matrix components are active regulators of this process. Collagen VI supports satellite-cell self-renewal and regeneration, demonstrating that niche composition can affect stem-cell behavior and is not merely structural support [96]. The specific molecular ligand, matrix organization, and bulk stiffness must still be distinguished because changing one matrix component can alter all three.

Aging makes that distinction especially important. Loss of fibronectin from the aged satellite-cell niche reduces regenerative capacity, and restoration of the relevant niche signal improves stem-cell function in mice [97]. Aged muscle stem cells also show cell-autonomous activation of p38 MAPK that compromises self-renewal [98]. Neither result is an actin-specific mechanism, yet both pathways intersect adhesion, polarity, and force transmission. They provide plausible routes by which an aged niche could alter MRTF or YAP/TAZ activity, but direct pathway measurements are required before that connection is stated as causal.

Fibrosis and chronic injury typically change matrix density, crosslinking, ligand availability, and tissue-scale loading at the same time. A stiff fibrotic substrate may increase cell spreading and YAP activity in one setting while restricting migration, fusion, or perfusion in another. In vitro hydrogels that vary only elastic modulus are valuable for isolation of one variable, but they cannot reproduce the topology or viscoelasticity of injured muscle. Conversely, tissue phenotypes cannot identify which mechanical property a cell sensed. Combining tunable matrices with ex vivo fibers and inducible in vivo perturbations offers a tractable hierarchy of evidence.

Age-associated reductions in SRF and MRTF-A suggest that actin-responsive transcription may decline in old muscle [58]. Muscle-specific SRF loss produces premature aging-like pathology, while myofiber SRF also controls overload-induced hypertrophy through paracrine signals that regulate satellite-cell proliferation and fusion [99,100]. These genetic models establish functions for SRF in both myofibers and their regenerative environment, although they do not assign every phenotype to G-actin-dependent MRTF signaling. Persistent YAP may support early progenitor expansion but impede timely differentiation, making pathway timing as important as mean activity.

Muscle atrophy should be distinguished from regenerative failure and age-related sarcopenia. In denervation and fasting models, desmin intermediate filaments disassemble before extensive myofibril breakdown, and experimentally disrupting desmin accelerates loss of actin and myosin. Suppression of tripartite motif-containing 32 (TRIM32), or of the paired box 4 (PAX4) and p97 pathway, attenuates this process [101]. YAP also has dose- and context-dependent effects in mature fibers. Endogenous YAP supports basal fiber size and is induced after denervation, whereas prolonged expression of constitutively active YAP produces atrophy and myopathy [102,103]. These findings link cytoskeletal organization and mechanosensitive transcription to muscle mass, but they concern mature-fiber proteostasis rather than the actin-dependent fusion mechanisms that build the syncytium.

Fusogen failure provides a distinct explanation for poor repair in degenerating muscle. Myomaker and Myomerger are induced in differentiating myogenic cells and act at separable membrane-remodeling steps [86,87,88,89,90,91]. This requirement is not confined to development. Deletion of Myomaker in adult satellite cells abolishes regeneration and leaves severe muscle destruction after injury [104], and in dystrophic mice the direction of the effect depends on the compartment, because deleting Myomaker in satellite cells worsens disease whereas reducing it in mature myofibers improves function and lowers damage [105]. In humans, hypomorphic MYMK alleles that reduce rather than eliminate fusogen function cause a congenital myopathy [106]. In a dystrophic or chronically injured environment, low abundance, incorrect surface delivery, or failed actin-dependent engagement of these fusogens could therefore restrict myonuclear accretion even when lineage commitment is preserved. Conversely, increased fusogen expression cannot be assumed to rescue defects in adhesion, protrusive force, or receiving-cortex mechanics. Injury studies should therefore quantify fusogen abundance and localization together with actin-rich contact formation, pore opening, and fiber repair.

The strongest near-term application is therefore not to label sarcopenia, muscular dystrophy, or muscle atrophy as an ‘actin-signaling disease.’ It is to identify which actin-dependent step fails in a defined context. A regenerative defect may arise from poor adhesion and migration, an altered MRTF–SRF program, mistimed YAP/TAZ activity, failure of contact-restricted branched actin and fusion, or impaired fusogen deployment. Atrophy may instead reflect loss of cytoskeletal support for mature myofibrils and altered mechanosensitive control of protein balance. These alternatives predict different rescue strategies. A global actin drug is unlikely to separate them and may disrupt mature sarcomeres. Pathway- and stage-specific interventions are more plausible.

## 8. An Integrated Model and Discriminating Experiments

### 8.1. Three Scales of Actin-Dependent Control

The evidence supports a model with three coupled but separable scales. At the molecular-pool scale, G-actin binding directly regulates MRTF transport and SRF coactivation. At the cell-mechanical scale, actomyosin tension, adhesion, geometry, and nuclear mechanics influence YAP/TAZ and other mechanosensitive outputs. At the subcellular scale, a branched actin network generates protrusive force at selected fusion contacts. Each scale has a characteristic measurement. Biochemical or genetically encoded monomer sensors for the first, traction or tension measurements for the second, and live super-resolution or correlative membrane imaging for the third.

Crosstalk is expected but should be demonstrated, not presumed. Polymerization can reduce free G-actin and simultaneously increase a filament scaffold for myosin, thereby activating MRTF and changing YAP/TAZ. Indirect mutual dependence of MRTF–SRF and YAP–TEAD through cytoskeletal dynamics has been demonstrated in cancer-associated fibroblasts [75], but equivalent coupling has not been established in myogenic cells. Filament stabilization, contractile bundles, and local Arp2/3 networks can also change independently. The model therefore predicts partial correlation, not identity, among MRTF activity, YAP/TAZ localization, traction force, and fusion.

This framework also clarifies the proliferation-to-differentiation transition. Early adhesion and mechanical signaling can sustain a proliferative YAP program while actin-dependent MRTF–SRF output primes structural genes. As cells reach appropriate density and receive differentiation cues, YAP must be attenuated, TAZ–MyoD and MRTF–SRF outputs can support commitment, and actin is repurposed for alignment and fusion. Current data do not establish one universal temporal sequence, and species, substrate, serum withdrawal, and injury state can shift the relative timing.

### 8.2. Measurements That Separate Mechanism from Correlation

Several recurring interpretations can be tested more rigorously. First, total phalloidin intensity should be paired with G/F fractionation or a live actin-state sensor and with spatial analysis. Second, nuclear MRTF or YAP should be paired with pathway-specific transcriptional output. Third, fusion should be decomposed into differentiation, contact, protrusion, lipid mixing, pore opening, and syncytium growth. Fourth, mechanical experiments should vary stiffness independently of ligand density and cell geometry. Table 2 summarizes what common measurements support and where the inference becomes too strong.

Acute, reversible perturbations are particularly useful because differentiation changes the cytoskeleton over hours to days. Optogenetic recruitment of Rho-family regulators or Arp2/3 activators can alter a network at a defined time and location, while degron-based depletion can separate requirements before commitment from those at fusion. Endogenous MRTF, YAP/TAZ, Myomaker, and Myomerger reporters can then reveal pathway order and reversibility within the same cell.

Epistasis provides the clearest test of pathway order. Actin-insensitive MRTF can test whether an actin regulator acts mainly through MRTF–SRF, and a TEAD-binding-deficient YAP mutant can separate YAP localization from YAP–TEAD output. At the fusion site, fusogen expression should not rescue a defect in invasive protrusion if actin-generated force is independently required.

Model choice should match the claim. C2C12 cells are appropriate for pathway dissection and high-content temporal imaging. Primary murine myoblasts retain genetic and injury-model relevance but are activated by isolation. Primary human myoblasts test donor and species relevance but introduce substantial biological variability. Primary embryonic chicken myoblasts are useful for direct imaging of myofibrillogenesis and fiber growth [12], while engineered 3D human muscle restores alignment, matrix geometry, and spontaneous contractile activity that are absent from conventional two-dimensional culture [13]. Isolated myofibers retain part of the satellite-cell niche, and inducible mouse genetics can define cell autonomy during regeneration. Drosophila remains valuable for resolving synapse architecture and force production. A persuasive mechanism need not use every model, but it should include at least one experiment that preserves physiological geometry and one that enables precise perturbation.

Two predictions are especially discriminating. The first is that matched increases in F-actin produced by branched versus contractile networks will generate different MRTF and YAP/TAZ outputs. The second is that contact-restricted Arp2/3 activation will rescue protrusion and fusion in a network-specific defect without normalizing whole-cell MRTF or YAP activity. Failure of these predictions would argue that the proposed scales are more tightly coupled than the current evidence suggests. Either outcome would improve the model because it specifies what would count against it.

## 9. Conclusions

Actin contributes to skeletal myogenesis through more than structural support. The G-actin pool directly controls MRTF–SRF signaling, actomyosin tension and adhesion influence YAP/TAZ in a stage-dependent manner, and localized branched networks generate force at the fusogenic synapse. These mechanisms overlap but cannot be inferred from one another. Treating actin abundance, turnover, tension, and location as separate variables explains why apparently similar perturbations can produce different effects on proliferation, differentiation, and fusion.

The field can now move from descriptive cytoskeletal phenotypes to causal, scale-resolved tests. Live reporters, spatially restricted perturbations, pathway-specific rescue, and conditional genetics can establish whether an actin change is instructive or a consequence of myogenesis. Such evidence is essential before actin-sensitive pathways are targeted in regeneration or age-related muscle decline. A mechanistic map that assigns each actin network to the decision it physically and transcriptionally controls is a more useful goal than a single universal actin switch.

## Figures and Tables

**Figure 1:**
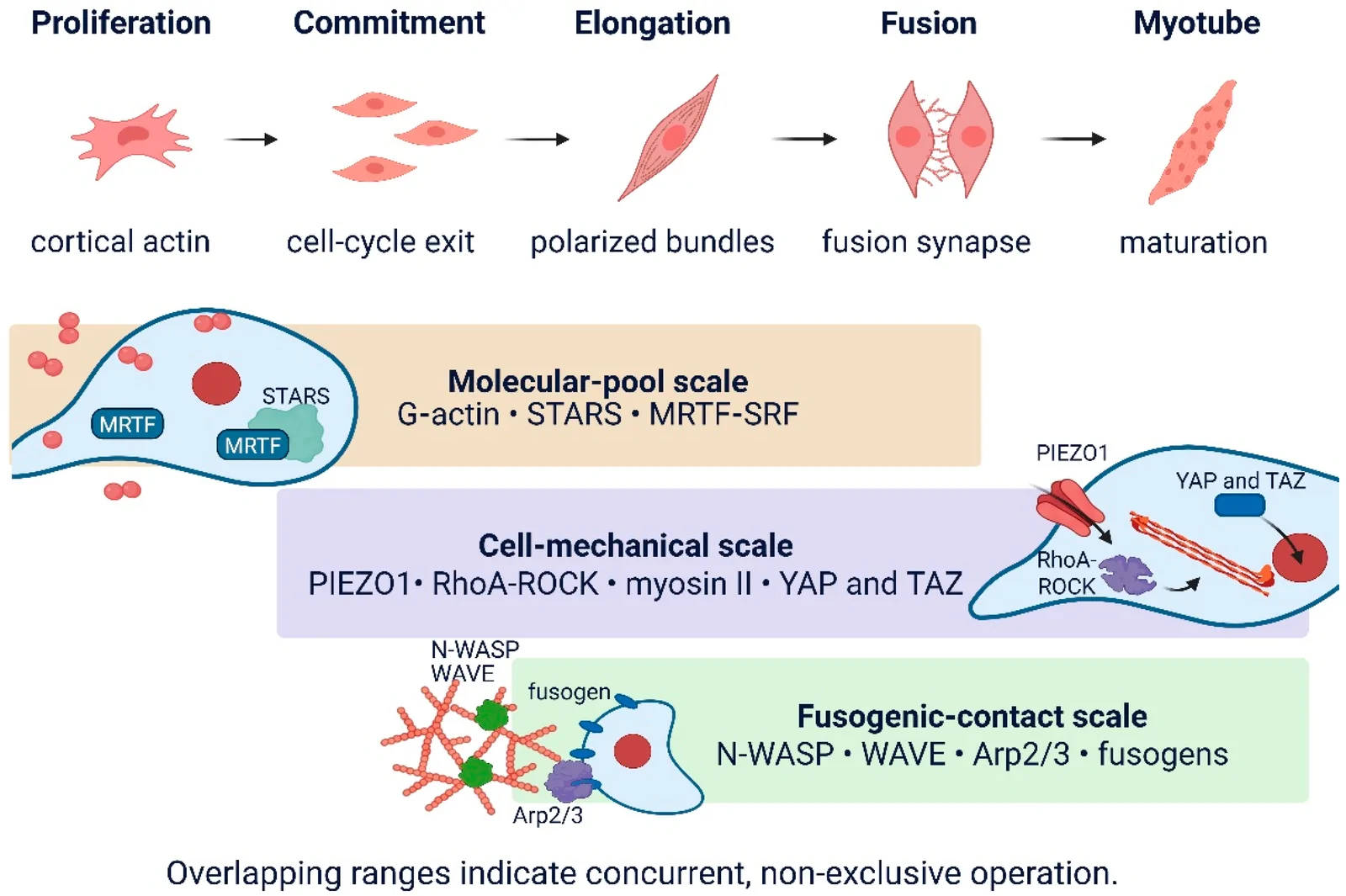
Stage-resolved actin functions in skeletal myogenesis. The horizontal bands indicate the stages during which the molecular-pool, cell-mechanical, and fusogenic-contact scales are most informative. Their overlap denotes concurrent, non-exclusive operation and does not imply a monotonic change in total F-actin. Components grouped within a scale do not necessarily form a single linear signaling pathway. Created in BioRender. Lee, W. (2026) https://BioRender.com/l8p88o7 (accessed on 21 September 2026).

**Figure 2:**
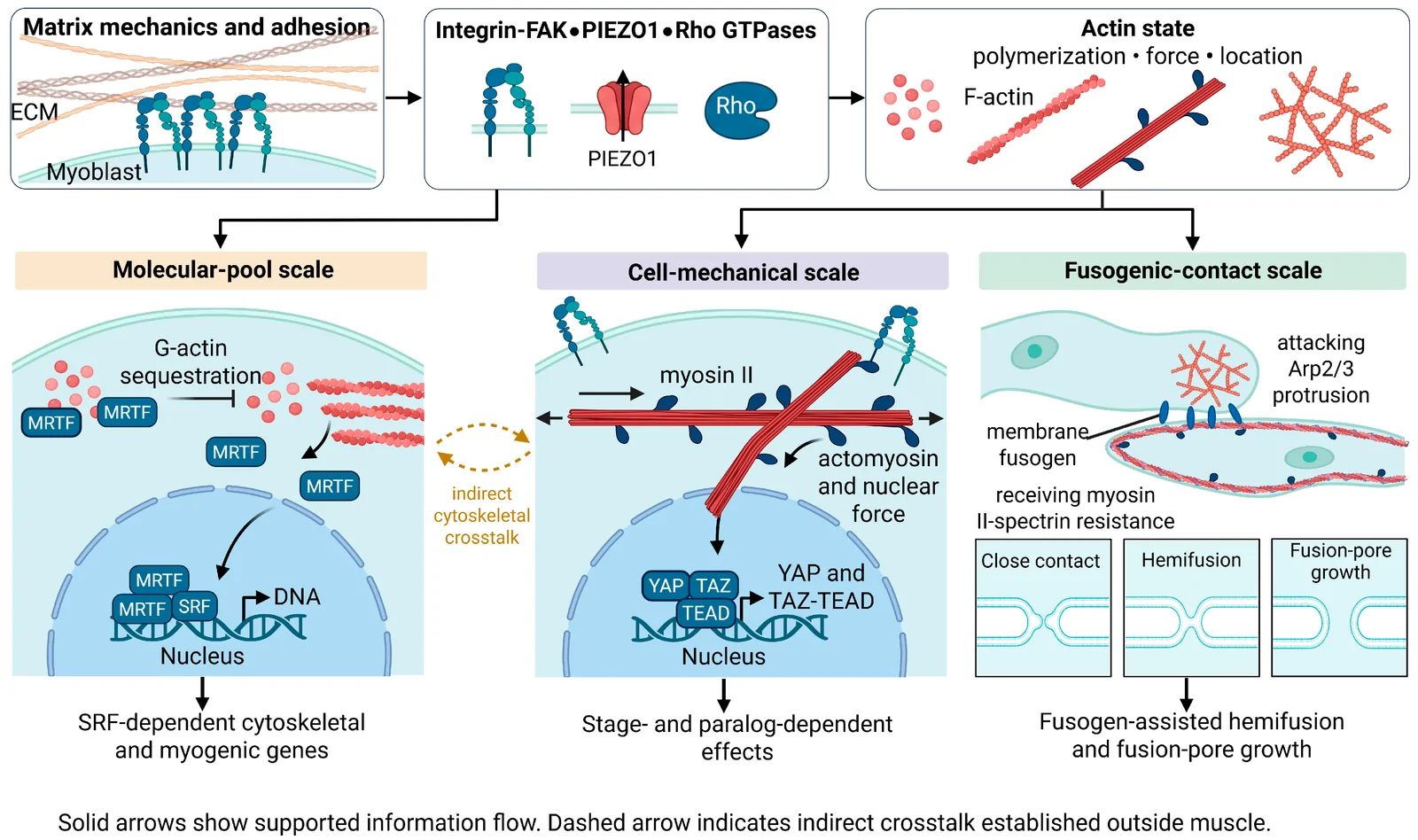
Integrated actin-dependent signaling and fusion model. Matrix and adhesion signals alter actin polymerization, tension, and localization. The available G-actin pool regulates MRTF-SRF. Actomyosin mechanics influence YAP and TAZ, and contact-restricted Arp2/3 networks cooperate with receiving-cell myosin II-spectrin resistance and muscle fusogens. Solid arrows denote supported information flow. The dashed bidirectional arrow denotes indirect cytoskeleton-mediated crosstalk between MRTF-SRF and YAP-TEAD demonstrated outside muscle, not a direct molecular interaction [41,42,75]. The attacking-receiving force asymmetry depicted in the fusogenic-contact panel is established primarily in Drosophila and has not been shown to be a stable organization in mammalian myoblast fusion. Created in BioRender. Lee, W. (2026) https://BioRender.com/lal69ao (accessed on 21 September 2026).

**Table 1:** Representative perturbation studies that assign actin regulators or associated signaling modules to defined myogenic outcomes.

Regulator or Module	Model Class and Perturbation	Direct Readout	Supported Conclusion	Interpretive Boundary
RhoA–mDia1	C2C12 gain- and loss-of-function	MyoD expression; cell-cycle progression; SRF dependence	mDia1 links actin-regulatory signaling to MyoD and proliferation [33].	SRF-dependent and independent outputs coexist.
STARS/ABRA–RhoA	C2C12 gain-of-function and SRF reporter assays	Actin polymerization, MRTF nuclear accumulation, and SRF activity	STARS couples a muscle-enriched actin regulator to MRTF–SRF signaling [34].	Some outputs may also involve MRTF-independent Rho signaling.
MRTF-A/MRTF-B–SRF	Cultured mouse myoblasts; muscle-specific mouse knockout	Differentiation, survival, muscle growth, sarcomere organization	The module is required for myogenic differentiation and muscle development [5,6].	SRF also receives non-MRTF inputs; late defects do not localize the initiating step.
Integrin–FAK	C2C12 pharmacologic and genetic perturbation	FAK activation; MyoD-related differentiation	Dynamic FAK signaling supports a promyogenic adhesion pathway [29,30].	FAK is pleiotropic and is not a direct measure of actin tension.
LINC complex–nesprin-1α2	Migrating myoblasts; in vivo mouse skeletal-muscle genetics	Nuclear positioning, centrosome orientation, and postnatal viability	The LINC complex provides a muscle-relevant physical route from cytoskeleton to nucleus [27,28].	These studies do not by themselves assign an MRTF- or YAP/TAZ-dependent transcriptional output.
N-WASP	Primary mouse myoblasts; conditional mouse deletion; cell-mixing assays	Differentiation, motility, attachment, and membrane fusion	N-WASP is selectively required for fusion and functions in both fusion partners [35].	The precise membrane-remodeling action still requires direct visualization.
Arp2/3 with N-WASP/WAVE	Primary satellite-cell-derived mouse myoblasts; conditional deletion during in vivo regeneration	Proliferation, differentiation, migration, protrusion, and fusion	Branched actin directly supports invasive protrusions and fusion [36].	Adult injury does not encompass all developmental fusion contexts.
Nap1	Mammalian cultured-myoblast depletion	Actin remodeling and fusion	Nap1-dependent remodeling is necessary for myoblast fusion [37].	Cell-culture depletion may combine contact, signaling, and fusion effects.
CAP2 and cofilin-2	Mouse knockout muscle	Myofibrillar actin differentiation, filament length, muscle architecture	Actin turnover proteins maintain late myofibrillar organization [20,22].	Mature structural phenotypes do not prove an early cell-fate mechanism.
MICAL2	Myogenic-cell loss of function	Lineage commitment and actin organization	MICAL2 is required for efficient commitment [38].	Redox and cytoskeletal outputs need catalytic rescue and in vivo separation.
Rac1–Cdc42–Dock1	In vivo mouse genetic models	Migration, morphology, and fusion	Rac1, Cdc42, and Dock1 are necessary for efficient mammalian fusion [39,40].	A fusion-index defect alone cannot distinguish pre-contact from pore formation.
Receiving-cell Myosin II–spectrin	Drosophila genetic perturbation, force sensing, and live imaging	Cortical tension and fusion-pore formation	The receiving cortex supplies resistance to invasive actin protrusions [41,42].	Stable partner asymmetry has not been established across mammalian fusion contexts.
PIEZO1–RhoA–ROCK	C2C12 cells; primary human myogenic cells	Calcium influx, lateral cortical actomyosin, fusion, and elongation	PIEZO1 organizes actomyosin to restrain excess fusion and support polarized myotubes [31].	The relevant output is controlled morphogenesis rather than maximal fusion.
Actin isoform assembly and YAP in avian primary cultures	Primary embryonic chick myoblasts and myotubes, actin isoform immunolocalization and microinjection, YAP activation	Sarcomeric versus cytoplasmic actin assembly, myofiber size	Avian primary cells support conserved roles in myofibrillogenesis and YAP-dependent growth [12,23].	Embryonic avian culture does not reproduce adult mammalian regeneration.
3D matrix architecture	Primary human or pluripotent-stem-cell-derived myoblasts in engineered 3D myobundles	Alignment, maturation, sarcomere organization, and spontaneous contraction	3D systems reproduce geometry and mechanical loading that are absent from conventional 2D culture [13].	Most constructs lack the complete neural, vascular, immune, and systemic niche.

Abbreviations: 3D, three-dimensional; ABRA, actin-binding Rho-activating protein; CAP, cyclase-associated protein; FAK, focal adhesion kinase; LINC, linker of nucleoskeleton and cytoskeleton; MRTF, myocardin-related transcription factor; N-WASP, neuronal Wiskott–Aldrich syndrome protein; PIEZO1, piezo-type mechanosensitive ion channel component 1; ROCK, Rho-associated coiled-coil-containing protein kinase; SRF, serum response factor; STARS, striated-muscle activator of Rho signaling; WAVE, WASP-family verprolin-homologous protein.

**Table 2:** Measurement-to-inference boundaries for studies of actin-dependent myogenic signaling.

Measurement	What It Directly Supports	Inference It Does Not Establish	Discriminating Addition	Representative Evidence
Phalloidin intensity or F/G fraction	Relative filament abundance or bulk partitioning at the sampled time	Filament turnover, tensile load, or productive local assembly	Live turnover sensor, fluorescence recovery after photobleaching (FRAP)/speckle imaging, traction measurement, and spatial segmentation	Actin pool control of MRTF [7,43]
Nuclear MRTF-A	Redistribution consistent with reduced G-actin occupancy	SRF target activation or causal control of differentiation	SRF reporter/occupancy, target genes, and rescue with actin-insensitive MRTF	G-actin mechanism [7,43,44]; import and mechanical redistribution [32,45]
Nuclear YAP or TAZ	Subcellular redistribution under the tested condition	TEAD-dependent transcription or a shared YAP/TAZ outcome	Isoform-specific perturbation, TEAD-binding mutant, and target-gene panel	Distinct YAP and TAZ effects [62,64]
Substrate elastic modulus	Response to the complete matrix system used	A stiffness-only effect independent of ligand density, porosity, and geometry	Orthogonal matrix chemistries with matched ligand presentation and cell area	C2C12 and muscle-stem-cell studies [10,25]
Differentiation markers	Entry into or progression through a molecular differentiation program	Direct membrane fusion or functional contractility	Single-cell lineage tracking, fusion-pore assay, and contractile readout	Cell-cycle exit can be uncoupled from differentiation [15]
Fusion index	Fraction of nuclei within multinucleated myotubes at an endpoint	The failed step, whether survival, alignment, contact, protrusion, lipid mixing, or pore growth	Stage-resolved live imaging with viability, migration, and protrusion controls	Synaptic tension and conditional branched-actin genetics [36,41,42]
Whole-cell knockdown of an actin regulator	Requirement of the protein for the composite phenotype	The relevant subcellular pool or a single downstream pathway	Localization-restricted rescue, acute degradation, and epistasis	Mammalian C2C12 signaling [55]; Drosophila fusion-site activity [78]

## Data Availability

No new data were created or analyzed in this study. Data sharing is not applicable to this article.
